# Left Atrial Dysfunction in Children with Repaired Pulmonary Artery Atresia with Ventricular Septal Defect: A Cardiovascular Magnetic Resonance Imaging Study

**DOI:** 10.3390/children9101536

**Published:** 2022-10-08

**Authors:** Yanyan Ma, Liwei Hu, Qian Wang, Aimin Sun, Rongzhen Ouyang, Jinglei Wang, Hao Zhang, Haibo Zhang, Chen Guo, Yumin Zhong

**Affiliations:** 1 Department of Radiology, Shanghai Children’s Medical Center, School of Medicine, Shanghai Jiao Tong University, Shanghai 200120, China; 2 Department of Cardiothoracic Surgery, Shanghai Children’s Medical Center, School of Medicine, Shanghai Jiao Tong University, Shanghai 200120, China

**Keywords:** cardiac magnetic resonance, congenital heart diseases, pulmonary artery atresia with ventricular septal defect, left atrium, strain

## Abstract

(1) Background: The left atrium (LA) is much more than a reservoir for left ventricular filling. The aim of this study was to assess the LA volume and function in patients with repaired pulmonary artery atresia with ventricular septal defect (rPA/VSD) using CMR. (2) Methods: 31 pediatric patients with rPA/VSD and 30 healthy controls were prospectively recruited. Left atrial ejection fraction (EF), strain and strain rate of three phases (reservoir, conduit, and pump) and left atrial volume were measured with cardiac function analysis software. (3) Results: Patients with rPA/VSD had decreased maximal volume index (*p* = 0.008). Compared to controls, LA reservoir strain and strain rate, conduit strain and strain rate, booster pump strain rate, total EF and passive EF were significantly lower (*p* = 0.001, *p* < 0.001, *p* = 0.001, *p* = 0.02, *p* = 0.03, *p* < 0.001, *p* < 0.001); the patients with preserved but lower RVEF(<50%) had lower reservoir strain, reservoir strain rate and pump strain rate (*p* = 0.01, *p* = 0.02, *p* = 0.04, respectively) than the patients with higher RVEF (≥50%). (4) Conclusions: In patients with rPA/VSD, LA function was altered when biventricular EF was preserved, which may provide an early indication of left ventricular diastolic dysfunction. CMR can detect LA dysfunction at an early stage, even before LA enlargement.

## 1. Introduction

Congenital heart diseases (CHD) is the most common birth defect, with approximately 122.7 per 100,000 live births affected by a CHD of the right heart structures. [1]. Pulmonary artery atresia with ventricular septal defect (PA/VSD) is a complex heart defect with an incidence of 4.2 to 10 per 100,000 live births. In this patient population, there is a wide spectrum of anatomical structures due to the size of central pulmonary arteries and pulmonary blood supply [2]. Common complications during following up after surgery include pulmonary artery residual obstruction and pulmonary regurgitation (PR), which can affect biventricular function and then lead to poor prognosis [3,4,5]. Therefore, regular follow-up and monitoring ventricular function is particularly important.

The role of left atrium (LA) in cardiac function is receiving increased attention [6]. LA is not just a reservoir for left ventricular filling. It has three functions during the cardiac cycle, including reservoir function, conduit function, and booster pump function. During the mitral valve closure, the LA receives the blood from the pulmonary veins. In early diastole, the LA delivers blood to the left ventricle (LV) in a passive way. Then in late diastole, the LA contracts, contributing about 15% of LV stroke volume. Emerging data demonstrated that LA volume and function can act as an early marker of LV dysfunction, even when LV ejection fraction is normal [6,7]. It also has been reported to be an important predictor, increasing the risk of adverse events in atrial fibrillation [8], hypertrophic cardiomyopathy [9], acute myocardial infarction [10], and heart failure [11,12].

Cardiac Magnetic Resonance Imaging (CMR) is considered as the gold standard for assessing atrial function compared with echocardiography [6,13]. Therefore, left atrial size and function assessed by CMR are expected to be useful indicators for evaluating cardiac function and prognosis during regular postoperative follow-up. Additionally, ventricular and atrial function of some postoperative right-sided malformations of CHD has been reported, such as pulmonary stenosis and tetralogy of Fallot (TOF). However, studies focusing on the atrial function in repaired PA/VSD (rPA/VSD) are lacking. Hence, the aim of our study was to analyze the left atrial function by CMR in patients with rPA/VSD.

## 2. Materials and Methods

### 2.1. Study Population

Thirty-one consecutive patients with rPA/VSD were recruited with CMR examination between August 2017 and February 2022. The inclusion criteria were as follows: (1) patients who had undergone repair with no residual shunting and no more than mild and moderate right ventricular outflow tract and pulmonary obstruction, (2) patients with preserved biventricular ejection fraction (LVEF ≥ 50%, RVEF ≥ 45%). The patients with (1) residual VSD, (2) decreased ventricular ejection fraction (LVEF < 50%, RVEF < 45%), (3) inadequate CMR image quality for assessment, and (4) CMR contraindications were excluded.

The control group of 30 gender- and age-matched children were selected from healthy volunteers. They had no abnormalities in cardiac function by echocardiography and no known history of cardiovascular diseases. In the healthy group, no contrast agent was used. The study was consistent with the Declaration of Helsinki and was approved by the institutional ethics committee. Informed consent was obtained from the guardians of all children involved in the study.

### 2.2. CMR Acqusitions

All patients underwent cardiac MRI performed with a 3.0 T MRI scanner (Discovery 750, GE Healthcare) or 1.5-Tesla (Achieva, Philips Healthcare, Best, The Netherlands) with an 8-channel phased array cardiac coil. Use of sedation depended on the patients’ status. If subjects were younger than five years old or could not be cooperative during the examination, sedation was carried out. Two-dimensional cine b-SSFP images were obtained with retrospective electrocardiograhic gating during free breathing or breath holding if they were able to cooperate, including short-axis views that cover both ventricles, two- and four-chamber views. Imaging parameters: echo time/repetition time 1.5–2.0/3.1–3.9 ms, slice thickness/interslice gap = 5–8/0 mm, temporal resolution/spatial resolution 25–32 ms/1.3–1.6 mm, and 20–30 reconstructed cardiac phases per cardiac cycle.

PR fractions of the main pulmonary artery (MPA) were calculated on two-dimensional phase contrast cine. Anatomical location of scanning was perpendicular to the MPA and slightly above the pulmonary valve with parameters including repetition time/ECG time 3.6–4.6 ms/3.3–4.6 ms, slice thickness 4–5 mm, and encoding velocity 120–300 cm/s.

#### 2.2.1. Biventricular Function and PR

We used the commercial cardiovascular post-processing CVI 5.9.1 software (Circle Cardiovascular Imaging, Calgary, AB, Canada) for assessing ventricular function and PR. Ventricular parameters were measured and indexed to the body surface area (BSA), including end-diastolic volume index (EDVi), end-systolic volume index (ESVi), stroke volume index (SVi), ejection fraction (EF), and cardiac output index (CI) of the left and right ventricle. PR fractions = regurgitant volume/forward flow volume

#### 2.2.2. Left Atrial Volumetric Analysis

**Figure 1:**
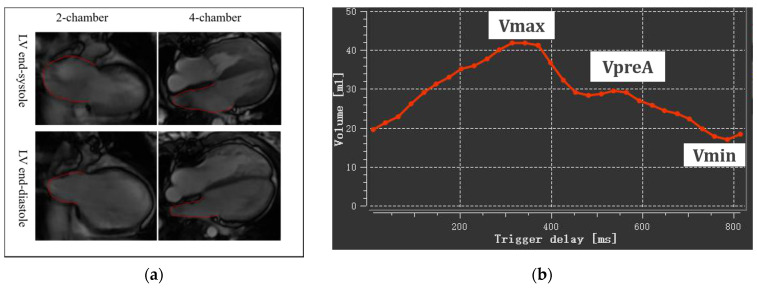
Left atrial volumetric analysis. (**a**) LA endocardium delineated on two-chamber and four-chamber views at LV end-systole and end-diastole; (**b**) A time-volume curve obtained by biplane area length method Vmax, maximal LA volume at LV end-systole; VpreA, LA volume at LV diastole before LA contraction; Vmin, minimal LA volume at LV end-diastole.

The assessment was performed on QMass 8.2 software (Medis BV, Leiden, The Netherlands) on the basic four-chamber and two-chamber cine views by biplane area-length method, including maximal LA volume at LV end-systole (LAVmax), LA volume at LV diastole before LA contraction (LAVpreA), and minimal LA volume at LV end-diastole (LAVmin) (Figure 1). All LA volumes were indexed to BSA: LAVImax, AVIpreA and LAVImin.

With the measured volumes above, we then calculated the LA ejection fraction (LAEF) at three phases. Total LAEF, passive LAEF, and active LAEF represented atrial reservoir, conduit, and pump function, respectively. The corresponding formulas were as follows:

Total LAEF = (LAVmax-LAVmin)/LAVmax × 100%

Passive LAEF = (LAVmax-LAVpreA)/LAVmax × 100%

Active LAEF = (LAVpreA-Vmin)/LAVpreA × 100%

#### 2.2.3. Left Atrial Strain and Strain Rate

**Figure 2:**
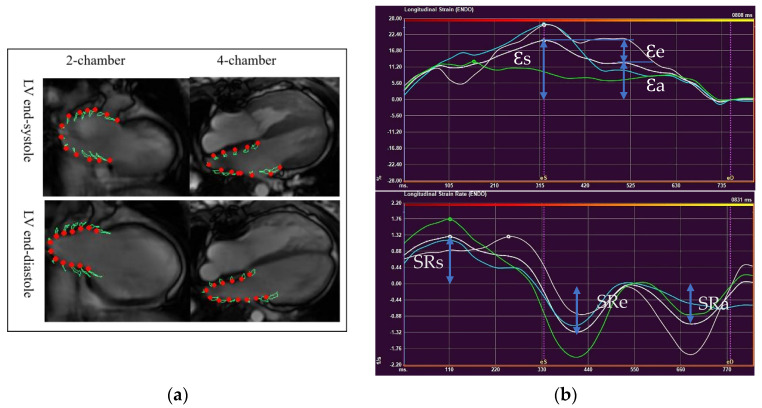
Left atrial deformation analysis using CMR-Feature Tracking. (**a**) LA endocardium delineated on two-chamber and four- chamber views at LV end-systole and end-diastole (**b**)A time-strain curve (**upper**) and a time-strain rate curve (**lower**) (Ɛs, LA reservoir strain; Ɛe, LA conduit strain; Ɛa, LA pump strain; SRs, LA reservoir strain rate; SRe, LA conduit strain rate; SRa, LA pump strain rate).

LA deformation analysis was performed with QStrain software (Medis BV, Leiden, The Netherlands), using two-and four-chamber views (Figure 2a). LA endocardium was well delineated in both views to track the atrial motion. The pulmonary veins and LA appendages were not included. Strain and strain rate curves were obtained from each view (Figure 2b). Final LA strain and strain rate (SR) values were the averages from two views, which correspond to three phasic functions.

### 2.3. Statistical Analysis

All statistical analyses were performed using commercially available SPSS 26.0 Statistics software (IBM, Armonk, NY, USA). The Kolmogorov-Smirnov test was used to assess the normal distribution of the data. Continuous variables were presented as mean ± standard deviation or median and interquartile range as appropriate. Categorical variables were stated as frequencies or percentages. If the continuous variables were normally distributed, comparisons between the two groups were performed using an independent-samples *t*-test. If the continuous variables were not normally distributed, the Mann-Whitney U test was used. Correlations between left atrial volume and strain parameters were assessed using Pearson’s correlation coefficient for normally distributed variables and Spearman’s correlation coefficient for non-normally variables. Correlations can be classified as weak, moderate, and strong, depending on the range of coefficients (<0.4, between 0.4 and 0.6, and >0.6, respectively). Based on 20 randomly selected subjects (10 controls and 10 patients with rPA/VSD), reproducibility was assessed by the intraclass correlation coefficient. A *p* value of ≤0.05 was considered statistically significant.

## 3. Results

### 3.1. Participants Information

Thirty-one consecutive patients with rPA/VSD and 30 volunteers were finally enrolled in the study. Baseline characteristics are showed in Table 1. The mean follow-up years after repair was 9.79 ± 3.49. There were no statistically significant differences in age, gender, and heart rate between the two groups. The controls had a slightly higher BSA. All patients were in sinus rhythm.

### 3.2. Ventricular Volumes and Function Characteristics

Left and right ventricular volume and function parameters in the two groups are presented in Table 1. Compared with controls, rPA/VSD patients had significantly higher RV EDV index, ESV index, SV index and CI (all *p* < 0.001). Both LVEF and RVEF were lower in patients with rPA/VSD but in the normal range. All subjects with rPA/VSD presented pulmonary regurgitation and the mean value was 36.27%.

### 3.3. Left Atrial Volume and Function Parameters

Among three LA volume indexes, there were no statistically significant differences in LAVIpreA and LAVImin between the two groups. However, patients with rPA/VSD had significantly smaller LAVImax (*p* = 0.008). Compared to controls, the reservoir strain and strain rate, conduit strain and strain rate, total EF, and passive EF were significantly lower in rPA/VSD (*p* = 0.001, *p* < 0.001, *p* = 0.001, *p* = 0.02, *p* < 0.001, *p* < 0.001, respectively). As for LA booster pump function parameters, only booster pump strain rate showed significant differences (*p* = 0.03). The details of the values for left atrial volume and function parameters were summarized in Table 2.

Total EF presented strong correlation with LA reservoir strain (r = 0.74, *p* < 0.001, Table 3), moderate correlation with LA reservoir SR, conduit strain and conduit SR, (r = 0.43, *p* = 0.02; r = 0.50, *p* = 0.01, r = 0.41, *p* = 0.02, Table 3).

### 3.4. Right Ventricular and Left Atrial Function Analysis

**Figure 3:**
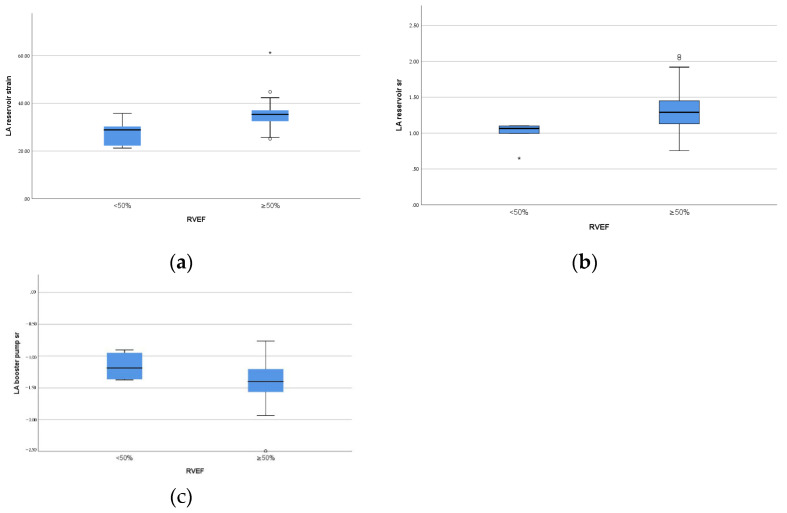
Box plots of left atrial function parameters with significant difference between different RV ejection fraction in the patients group. (**a**) LA reservoir strain; (**b**) LA reservoir strain rate; (**c**) LA pump strain rate.

As showed in Table 4 and Figure 3, in rPA/VSD group, the patients with preserved but lower RVEF (<50%, *n* = 6) had lower reservoir strain and SR and pump SR (*p* = 0.01, *p* = 0.02, *p* = 0.04, respectively) than the patients with higher RVEF (≥50%, *n* = 25).

### 3.5. Reproducibility of Left Atrial Function Parameters

The ICC of left atrial strain and SR were listed in Table 5. There was good inter- and intra-observer reproducibility for reservoir and conduit strain parameters, but the reproducibility of pump strain and SR was moderate.

## 4. Discussion

The present CMR study demonstrated the alteration of LA size and function in patients with rPA/VSD, characterized by diminished strain and strain rates at reservoir and conduit phase, and reduced booster pump SR before LA dilation. Furthermore, patients showed no increased left atrial volume but had decreased maximal volume index. An additional interesting finding was that the LA performed better in patients with higher RVEF (≥50%).

### 4.1. Right Ventricular Dysfunction in rPA/VSD Patients with Preserved EF

In our study, we found that rPA/VSD group with preserved ventricular EF had significantly impaired RV performance with higher RVEDVi, ESVi, SVi and CI compared with controls. On the other hand, all patients demonstrated pulmonary regurgitation, which is a common condition in postoperative right-sided malformations of congenital heart diseases. In the long run, it could cause irreversible ventricular remodeling and cardiac dysfunction [14]. These findings were consistent with previous studies [15]. Our explanation was that PR mainly contributed to RV dilatation. In the setting of chronic PR, pulmonary blood flowing backward in diastole would bring an increasing afterload of RV and then the ventricle underwent adaptive changes. Associations between PR and higher RV volume parameters had also been reported in other right-sided CHD [3,16].

### 4.2. Left Atrial Impaired Performance in rPA/VSD Patients with Preserved EF

Emerging data suggested that the LA is much more than a reservoir for LV filling. As previously mentioned, it has three phasic functions: (1) reservoir, (2) conduit, and (3) contractile function. Its structure and function are expected to be useful indicators for evaluating cardiac function and prognosis in multiple disease states, including CHD [5]. Moreover, deformation analysis by CMR-Feature Tracking is a more promising tool for the early detection of atrial dysfunction than atrial volumetric analysis.

To the best of our knowledge, this is the first study about the assessment of left atrial volume and function in rPA/VSD by CMR. In this group with the patients with preserved biventricular EF, we demonstrated significantly lower LA reservoir strain, reservoir SR, total EF, conduit strain, passive EF, and lower pump SR than those in healthy controls, indicating impaired reservoir, conduit and pump function. It is suggested that abnormal LA function might precede LV dysfunction, which may be an early indicator of LV dysfunction. Our findings were consistent with a previous echocardiographic study by Hou et al. [17] in patients with rTOF. Sometimes, PA/VSD can be considered as a different entity or a severe form of TOF with pulmonary stenosis [18]. They observed LA reservoir, conduit, and pump function impairments, and found impairments were associated with LV diastolic performance. As for contractile function, Hou et al. found impaired performance with significant lower pump strain and SR in longer follow-up time (14.4 ± 4.4 years), on the contrary, another CMR study of rTOF in shorter follow-up time (9.65 ± 3.65 years) showed better booster pump function [5]. A possible explanation for this is the different time of following up after repair. There might be a compensatory increase in contractile function of LA with a decrease in reservoir and conduit function during the early age to maintain adequate LV filling, but a decompensatory decrease in later life. This mechanism was in line with a previous study that also implied that this common pathway for atrial dysfunction was independent of the types of lesions in CHD [7]. Severity of the disease state and different modality may also make a contribution to the different performance of left atrial contractile function. Further comparative study is needed to assess the atrial mechanisms in rPA/VSD and rTOF. In addition, due to the lack of early postoperative data, the pattern of LA function alterations during the following up could not be identified. Hence, earlier and regular follow-up after repair is warranted in the future.

On the other hand, in the presence of abnormal LA function, children with rPA/VSD showed no increase in LA volume, but a decrease in LAVImax. This may be due to the reduced pulmonary blood flow secondary to PR, leading to a decrease in the preload and thus inadequate filling of the left atrium. Above all, it is suggested that abnormalities in left atrial function had already have occurred in children with rPA/VSD before the enlargement of the left atrium.

In addition, we also found moderate to good correlations between the strain parameters and total EF, which corresponds to the reservoir function of LA and can be simply calculated by LAVmax and LAVmin. This was consistent with previous studies in HCM [19] and non-cardiovascular diseases [20]. Alfuhied et al. also found that LA volume and EF had better reproducibility than strain parameters by CMR. Therefore, using total EF to assess LA function in clinical practice routinely may be more feasible in the short term compared with deformation analysis of LA.

### 4.3. Atrial-Ventricular Interactions

The left atrium is one component of the whole heart, which should not be separated from the other chambers. It interacts with the LV through the atrioventricular junction and manifests a dynamic adaptation in its size and function for left ventricular compliance alterations [7]. When LV filling behaves abnormally, these changes can be identified by deformation analysis, as mentioned above. Right atrial–right ventricular interaction has also been reported with positive correlation of right atrial and ventricular longitudinal strain [21]. However, there are limited data regarding the association between right ventricular function and LA performance, which can be reasonable due to the presence of ventricular-ventricular interactions [22]. The LV and RV share the ventricular septal wall [23]. RV dilation can prolong the duration of the free wall contraction, resulting in the septum shifting to the left and LV filling impairment [24,25]. With increased afterload, LA conduit and contractile function would be disturbed by reduced blood velocity in the mitral valve [26]. Then, LA compliance can be impaired in view of its close interaction with LA contraction in LA early filling [27]. Moreover, LA early reservoir function, which is mainly related to atrial compliance, would be diminished. This is consistent with Hou et al. [17] who found a negative correlation between LA conduit SR and RV volume overload. In our study, we analyzed LA performance in two different range of RVEF. The patients with higher RVEF (≥50%) had better reservoir function and booster pump function with significantly higher reservoir strain, reservoir SR, and pump SR compared to the patients with preserved but lower RVEF (<50%) in rPA/VSD. Combining the above findings, we postulated that there may exist in left atrial–right ventricular interactions. However, there were only six patients with RVEF (<50%). A future larger sample study is required to determine the interaction between LA and RV function and the intrinsic mechanisms.

### 4.4. Limitations

There were some limitations to this study. First, this was not a multi-center study and the number of the rPA/VSD is limited; thus, the data should be considered preliminary. Second, interactions between the atria have been reported [28], and the assessment of right atrial function could be added in the future. Third, two scanner types were employed, with only nine patients on the 1.5 T system. Furthermore, compared with echocardiography, lower temporal resolution of CMR may affect the measurement of SRs. Finally, Larger further cohorts are needed to investigate the role of left atrial size and function in patients with rPA/VSD and other postoperative CHD.

## 5. Conclusions

In patients with rPA/VSD, left atrial function has been altered when left ventricular ejection fraction was preserved, which may provide an early indication of left ventricular diastolic dysfunction. In addition, CMR can detect abnormalities of left atrial function at an early stage, even before left atrial enlargement.

## Figures and Tables

**Table 1:** Baseline demographic and biventricular function.

Variable	rPA/VSD	Control	*p*
Number	31	30	
Age	10.84 ± 3.62	11.06 ± 2.97	0.92
Female(%)	12(38)	10(33)	
Heart rate(beat/min)	74.50 ± 16	80.00 ± 20	0.14
BSA	1.14 ± 0.29	1.33 ± 0.31	**0.02**
Years after repair	9.79 ± 3.48		
QRS duration	109.71 ± 24.10		
Type of surgery			
complete repair	31		
cardiac intervention	14		
LVEDVI (ml/m^2^)	75.34 ± 15.60	76.58 ± 14.80	0.98
LVESVI (ml/m^2^)	33.72 ± 7.31	30.00 ± 6.45	0.05
LVSVI (ml/m^2^)	45.80 ± 10.91	48.68 ± 10.32	0.19
LVEF (%)	58.42 ± 3.56	62.93 ± 7.29	**0.005**
LVCI	3.54 ± 0.77	3.66 ± 0.81	0.48
RVEDVI (ml/m^2^)	114.39 ± 41.68	77.18 ± 21.67	**<0.001**
RVESVI (ml/m^2^)	50.29 ± 29.39	35.18 ± 10.32	**<0.001**
RVSVI (ml/m^2^)	63.87 ± 19.41	44.09 ± 18.15	**<0.001**
RVEF (%)	54.38 ± 8.54	55.02 ± 10.01	0.83
RVCI	5.15 ± 1.27	3.01 ± 0.66	**<0.001**
PR(%)	36.27 ± 14.02	/	

LVEDVI = left ventricular end-diastolic volume index; LVESVI = left ventricular end-systolic volume index; LVSVI = left ventricular stroke volume index; LVEF = left ventricular ejection fraction; LVCI = left ventricular cardiac output index; RVEDVI = right ventricular end-diastolic volume index; RVESVI = right ventricular end-systolic volume index; RVSVI = right ventricular stroke volume index; RVEF = right ventricular ejection fraction; RVCI = right ventricular cardiac output index; PR = Pulmonary Regurgitation fraction. Statistically significant differences were showed in bold.

**Table 2:** Left atrial volume and phasic function between rPA/VSD and healthy controls.

Variable	rPA/VSD	Control	*p*
LA volume parameters			
LAVImax	27.82 ± 5.37	31.84 ± 6.11	**0.008**
LAVImin	11.80 ± 2.87	11.06 ± 3.05	0.34
LAVIpre-A	17.33 ± 4.46	15.42 ± 5.65	0.34
LA reservoir function			
Strain(%)	34.77 ± 7.70	38.39 ± 4.76	**0.001**
Strain rate	1.27 ± 0.33	1.95 ± 0.37	**<0.001**
Total EF	0.58 ± 0.06	0.65 ± 0.05	**<0.001**
LA conduit function			
strain(%)	18.54 ± 6.75	24.03 ± 7.17	**0.001**
Strain rate	−1.74 ± 0.76	−2.15 ± 0.73	**0.02**
Passive EF	0.37 ± 0.08	0.52 ± 0.08	**<0.001**
LA booster pump function			
Strain (%)	15.15 ± 4.95	14.38 ± 5.43	0.56
Strain rate	−1.36 ± 0.32	−1.58 ± 0.54	**0.03**
Active EF	0.32 ± 0.10	0.28 ± 0.11	0.26

LAVImax: maximal LA volume at LV end-systole index, LAVImin: minimal LA volume at LV end-diastole index, LAVIpre-a: LA volume at LV diastole before LA contraction index. Statistically significant differences were shown in bold.

**Table 3:** Correlations between left atrial volume with strain parameters in the rPA/VSD.

Variable	Total EF	
	r	*p*
Reservoir strain	0.74	**<0.001**
Conduit strain	0.50	**0.01**
Pump strain	0.41	**0.02**
Reservoir SR	0.43	**0.02**
Conduit SR	−0.41	0.93
Pump SR	−0.17	0.36

SR: strain rate. Statistically significant differences were shown in bold.

**Table 4:** Left atrial function between different RV ejection fraction in the patients group.

Variable	RVEF (%)		*p*
	≥50 (*n* = 25)	<50 (*n* = 6)	
Reservoir strain	35.45 ± 7.26	27.90 ± 5.40	**0.01**
Reservoir SR	1.31 ± 0.35	0.99 ± 0.17	**0.02**
Conduit strain	19.81 ± 6.80	14.83 ± 7.76	0.88
Conduit SR	−1.8 ± 0.797	−1.43 ± 0.49	0.17
Pump strain	15.65 ± 4.62	13.06 ± 6/17	0.11
Pump SR	−1.42 ± 0.35	−1.16 ± 0.20	**0.04**

SR: strain rate. Statistically significant differences were shown in bold.

**Table 5:** Intra- and inter-observer reproducibility of left atrial function parameters.

Parameters	ICC	
	Interobserver	Intraobserver
Reservoir strain	0.92	0.96
Conduit strain	0.89	0.88
Pump strain	0.72	0.75
Reservoir SR	0.85	0.76
Conduit SR	0.92	0.86
Pump SR	0.73	0.72

SR: strain rate. ICC = intraclass correlation coefficients. Statistically significant differences were shown in bold.

## Data Availability

Not applicable.

## Abbreviations

CHD Congenital heart diseases; PA/VSD Pulmonary artery atresia with ventricular septal defect; rPA/VSD Repaired PA/VSD; PR Pulmonary regurgitation; LA Left atrium; LV Left ventricle; RV Right ventricular; CMR Cardiac Magnetic Resonance Imaging; TOF Tetralogy of Fallot; rTOF Repaired Tetralogy of Fallot; MPA main pulmonary artery;EDVi end-diastolic volume index; ESVi end-systolic volume index; SVi stroke volume index; EF Ejection fraction; CI Cardiac output index; BSA body surface area; LAVmax maximal LA volume at LV end-systole; LAVpreA LA volume at LV diastole before LA contraction; LAVmin minimal LA volume at LV end-diastole; Ɛs LA strain at reservoir; Ɛe LA strain at conduit; Ɛa LA strain at booster-pump phase; SRs LA strain rate at reservoir; SRe LA strain rate at conduit; SRa LA strain rate at booster-pump phase; SR strain rate; ICC intraclass correlation coefficients.
